# National

**Published:** 1897-04

**Authors:** 


*National.* The first official order issued by Secretary Wilson, of the Department of Agriculture, made its appearance on March 9th. It concerns the exportation of beef to foreign countries, and provides "that from and after March 15, 1897, all beef offered for transportation to European ports, whether fresh, salted, canned, corned, or packed, being the meat of cattle killed after the passage of the Act under which this order is made, shall be accompanied by a certificate issued by an inspector of the department showing that the cattle from which it was produced were free from disease and the meat sound and wholesome; and in order that it may be determined whether all beef exported to European ports has been so inspected and found to be wholesome, it is further ordered that the meat of all other species of animals exported to such ports which for any reason does not bear the inspection stamps of the department, shall be packed in barrels, cases, or other packages which are legibly marked in such manner as to clearly indicate the species of animal from which the meat was produced. Meat which is not so marked and which is not accompanied by a certificate of inspection will be classed as uninspected beef and will not be allowed exportation to European ports."

No clearance is to be given to any vessel having on board such meats until the provisions of the order are complied with. Until otherwise ordered, certificates will not be required with beef exported to other than European countries. The original order of the Secretary of Agriculture, of August 28, 1895, for carrying out the provisions of Section 2 of the Act under which the order is made was postponed to the date set out in Secretary Wilson's circular.

